# The Elite Sport Relevance Index: a five-dimensional framework for understanding value creation in elite sport

**DOI:** 10.3389/fspor.2026.1944243

**Published:** 2026-10-19

**Authors:** Jan-Willem Van der Roest, Bartel Berkhout

**Affiliations:** 1 Research Group Sport Management & Sport Business, Faculty of Health, Sports and Physical Activity, Amsterdam University of Applied Sciences, Amsterdam, Netherlands; 2 SportsInQ, Amsterdam, Netherlands; 3 Nyenrode Business University, Breukelen, Netherlands

**Keywords:** elite sport, elite sport relevance index, governance, institutional logics, leadership, legitimacy, sustainability, value creation

## Abstract

Elite sport has traditionally derived its legitimacy from competitive success and international prestige. However, growing expectations concerning social responsibility, sustainability, economic accountability, and emotional engagement increasingly challenge this performance-centric rationale. Sport management research has addressed these developments across disparate strands, but lacks an integrative framework that captures elite sport relevance as a multidimensional and dynamic process of value creation. This article introduces the Elite Sport Relevance Index (ESRI), a conceptual framework comprising five interrelated dimensions: Performance, People, Planet, Profit, and Passion. Building on the Triple Bottom Line and sport-specific value frameworks, the ESRI conceptualizes elite sport as a value-creating system operating across sporting, social, environmental, economic, and cultural-emotional domains. A focused synthesis of sport management literature, complemented by illustrative examples from elite sport practice, explicates each dimension and theorizes their interaction through institutional logics. The ESRI contributes a diagnostic and deliberative framework that makes complementarities, tensions, structural conflicts, and power-laden prioritizations visible. It positions leadership and governance as mediating but bounded mechanisms through which value claims are translated into material practices, while recognizing that not all contradictions can be resolved organizationally.

## 1 Introduction

Elite sport occupies a prominent yet increasingly contested position in contemporary societies. Historically, its relevance has been justified primarily through competitive success—medals, rankings, championships, and international prestige—and through a business logic linking success to on-field performance (1–4). These rationales have shaped policy, strategy, and investment, while the wider value of elite sport was often assumed rather than interrogated. That assumption is increasingly difficult to sustain. Governments, sponsors, media, communities, athletes, and fans now expect value beyond competitive outcomes, including athlete welfare, social responsibility, environmental sustainability, economic accountability, and emotional engagement (5–9).

In several publicly funded national elite sport systems, policy objectives have broadened beyond medal production to include athlete well-being, governance, sustainability, and societal impact. This is not a uniform transition: professional clubs, leagues, event owners, and other commercial actors have long pursued varying combinations of sporting success, revenue, audience attention, and institutional prestige. The Australian Sports Commission's Win Well strategy, for example, connects international success with athlete well-being and system sustainability, while UK Sport links performance to societal impact and responsible governance (10, 11). These shifts coincide with growing attention to burnout, mental health, and adjustment pressures among elite athletes (12). The challenge is therefore to sustain performance amid resource constraints, societal scrutiny, and competing value demands.

Sport management scholarship has increasingly engaged with value creation in elite sport through themes such as public value, corporate social responsibility, and sustainability. Although this work offers important insights, it remains analytically fragmented at the organizational level. Many studies focus on single value domains (13, 14), offering limited guidance on how elite sport organizations operate when confronted with multiple and often competing demands. Ecosystem-level approaches to sport value creation provide an important foundation (15), but they offer less leverage for examining how trade-offs are negotiated within individual organizations and systems. We therefore lack a coherent organizational framework that captures elite sport as an arena in which different values coexist, interact, and must be prioritized, accommodated, or left in tension.

This article addresses this gap by proposing the Elite Sport Relevance Index (ESRI), a conceptual framework structured around five interrelated value dimensions: Performance, People, Planet, Profit, and Passion. Rather than proposing a normative hierarchy of values, the ESRI makes explicit the multiple—and often competing—criteria through which elite sport is evaluated, governed, and legitimized. By conceptualizing elite sport as a value-creating system operating across sporting, social, environmental, economic, and emotional domains, the framework enables a more nuanced understanding of elite sport relevance in contemporary society. The central question is no longer how elite sport systems can become more efficient, but how they can be redesigned to remain relevant, legitimate, and sustainable in a changing world.

The terms elite sport and high-performance sport are both used in the literature to describe sport at its highest competitive levels. Sotiriadou and De Bosscher (16) employ high-performance sport as an umbrella term for the top end of sport development, encompassing athletes and teams competing at national or international level across Olympic, Paralympic, non-Olympic, professional, team, and emerging sports. Their conceptualization extends beyond competition itself to include the processes through which athletes are attracted, retained or transitioned, nurtured, and enabled to reach and sustain elite performance. High-performance sport is consequently understood as an open system in which developments at the micro level of athletes and coaches, the meso level of organizations and sport policy, and the macro level of social, cultural, economic, and political conditions interact. In this article, we use the term elite sport consistently while recognizing its substantial overlap with what Sotiriadou and De Bosscher call high-performance sport. We prefer elite sport because the ESRI addresses multidimensional value creation and its wider organizational and societal consequences, rather than focusing primarily on the athlete-development and performance-production processes often emphasized in high-performance sport management.

*Elite sport organizations* are consequently defined as formal entities whose core activities govern, support, organize, deliver, or commercialize elite sport. These include national sport agencies, Olympic and Paralympic committees, sport federations, high-performance institutes, professional clubs and leagues, and major-event organizations. Professional sport overlaps with elite sport but is not synonymous with it: professional status concerns remuneration and commercial organization, whereas elite status concerns the level of competition and performance. Individual athletes are central participants, stakeholders, and organizational actors, but the primary units of analysis in the ESRI are organizations and systems. Athlete-level material is included only where it illuminates these broader organizational or systemic arrangements.

## 2 Conceptual foundations

### 2.1 Elite sport as a value-creating domain

The ESRI framework builds on and extends established approaches to multidimensional value creation in management and organizational research. In this article, value creation refers to the processes through which organizational activities produce benefits and meaningful outcomes for stakeholders. Critical assessment of these processes must also consider the harms, costs, or losses imposed on stakeholders or the natural environment. The resulting consequences may be public or private and need not be exclusively economic or monetizable (17). The framework's conceptual roots can be traced to the Triple Bottom Line, which conceptualizes organizational value across economic, social, and environmental dimensions (18). Although influential, that framework does not fully capture the distinctive nature of value creation in elite sport, where competitive performance and emotional engagement are defining characteristics of the domain (19).

To address this limitation, the ESRI incorporates two additional dimensions that are central to sport as a social and cultural institution: Performance and Passion. Performance reflects the competitive excellence that defines elite sport and differentiates it from other sectors (2), while Passion captures the emotional, symbolic, and identity-based value generated through fan engagement, inspiration, and collective meaning-making (19). Together with People, Planet, and Profit, these dimensions form a comprehensive yet parsimonious framework for analyzing elite sport relevance.

### 2.2 Theoretical orientation and scope of the ESRI

The ESRI adopts a non-teleological, organizational and system-level perspective. It does not derive a universal hierarchy of value from an assumed intrinsic purpose of sport. Instead, it treats elite sport relevance as institutionally plural, context-dependent, contested and shaped by power. Performance is constitutive of elite sport, but this does not mean that it automatically takes precedence over the other dimensions. Equally, analytical parity between the five dimensions does not imply ethical equivalence or compensability: competitive or commercial achievements cannot simply offset serious human or environmental harm.

The ESRI is related to, but analytically distinct from, established frameworks such as SPLISS and socioecological models. SPLISS identifies national policy inputs and throughputs that increase opportunities for international sporting success through nine policy pillars (2). It therefore primarily explains how national elite sport systems produce Performance. The ESRI instead examines the multiple forms of value and harm through which organizations and systems are evaluated, including but extending beyond competitive outcomes. Socioecological models organize influences and consequences across nested levels (20), whereas ecosystem-level approaches emphasize networks and value co-creation (15). These frameworks can be used complementarily: socioecological models help locate where influences and consequences arise; SPLISS explains how national systems organize the production of sporting success; ecosystem approaches map actors and exchanges; and the ESRI diagnoses how value, harms, and trade-offs interact and are prioritized. The distinctive purpose of the ESRI is thus to expose contested value configurations across Performance, People, Planet, Profit, and Passion.

Importantly, the ESRI is not intended as a static categorization of value domains, but as an analytical lens that makes explicit how elite sport organizations and systems navigate multiple and often competing institutional logics (21–23). The five dimensions are not themselves institutional logics; they are analytical domains of value that are interpreted and enacted through competitive, market, state, professional, community, and environmental logics. These logics may reinforce one another, but they may also generate tensions that leaders and governance arrangements must prioritize, contest, accommodate, or leave unresolved. Making these dynamics explicit is central to understanding elite sport relevance under contemporary conditions of accountability and scrutiny.

The analytical inclusion of all five dimensions does not imply ethical equivalence or unrestricted substitutability. The ESRI is not an additive index in which gains in Performance, Profit, or Passion can automatically compensate for severe harm under People or Planet. Legal obligations, human rights, integrity requirements, and ecological limits may impose thresholds that constrain an acceptable configuration. The framework does not determine these thresholds in advance; rather, it makes visible when they require explicit ethical, political, or regulatory judgment. The absence of a universal ranking principle should therefore be understood as an invitation to transparent deliberation, not as permission to offset serious harm against sporting or commercial success.

### 2.3 Elite sport and public value creation

One prominent strand of sport management literature conceptualizes elite sport as a contributor to public value. This work positions elite sport not merely as a recipient of public investment, but as a societal domain through which collective goals, norms, and outcomes can be pursued and contested. Public value creation refers here to outcomes and processes that are collectively valued and benefit the public, together with the demonstration of those outcomes and processes (24, 25). Elite sport may contribute to public value through outcomes such as sport participation and health, social cohesion, national identity, and shared meaning (5, 8).

In earlier work, elite sport has been conceptualized as part of a broader public value ecosystem, in which value is not confined to measurable outputs but emerges through processes of interaction, interpretation, and societal embedding (9, 25). From this perspective, public value is inherently contextual and relational: elite sport may generate value differently depending on political priorities, cultural narratives, media framing, and institutional arrangements (26).

While these contributions have significantly advanced our understanding of elite sport as a source of public value, they offer limited guidance on how such value can be actively created, contested, and sustained in practice. In particular, they provide insufficient analytical leverage for leaders seeking to navigate tensions between competing value demands within elite sport systems. This limitation points to the need for a more integrative conceptual approach that clarifies how elite sport organizations and systems create public value while also recognizing harms, costs, and unresolved conflict.

### 2.4 Elite sport and value creation for private organizations

Alongside public value creation, elite sport generates private value for sponsors, media companies, commercial partners, employers, and other actors in the broader sport ecosystem. Private value refers here to economic, reputational, symbolic, relational, or experiential benefits appropriated by identifiable actors. As Andrews (27) observes, from the second half of the twentieth century—particularly in the United States—sport became increasingly integrated into the structures of consumer capitalism, evolving into a large-scale economic enterprise in which sporting institutions, events, teams, and athletes became deeply enmeshed with commercial logics, market relations, and commodity-oriented cultural production. This process of commercialization subsequently extended to other Western contexts and has now developed into a global phenomenon (28).

Within this context, elite sport functions as a platform for multiple, interconnected forms of value creation. Organizations derive economic, reputational, symbolic, and relational value through their association with elite sport, ranging from brand exposure and market positioning to stakeholder engagement and internal organizational benefits. These forms of value may reinforce one another over time, as sporting success, brand association, and emotional engagement jointly enhance financial outcomes (29).

Private value is created within interconnected networks of sport organizations, businesses, media, and consumers rather than through isolated transactions (14, 15). The ESRI therefore examines how economic, reputational, symbolic, and relational benefits interact with public consequences across the wider elite sport ecosystem.

Individual athletes may also act as entrepreneurial and media actors. Consistent with the ESRI's unit of analysis, such activity is considered only where it reshapes organizational relationships, governance, or the distribution of private value within elite sport systems (30).

## 3 The five dimensions of elite sport relevance

The preceding sections have shown that elite sport creates value across multiple public and private domains, and that these value processes are increasingly interconnected, dynamic, and context-dependent. At the same time, the literature offers limited conceptual tools to capture how these different forms of value relate to one another at the level of elite sport organizations and systems, and how they can be navigated in practice.

The concept of “relevance” provides the bridge between the preceding discussion of value creation and the ESRI framework. Building on stakeholder theory, which conceptualizes organizational value as multidimensional and dependent on what different stakeholders seek and experience (17), relevance refers here to the extent to which elite sport creates forms of value that stakeholders regard as meaningful and worthy of continued support. Legitimacy is related but distinct: it concerns whether an organization and its actions are perceived as appropriate within prevailing norms and expectations (31). Against this backdrop, the ESRI provides a structured lens through which multidimensional value creation and its associated harms and trade-offs can be analyzed across five interrelated dimensions: Performance, People, Planet, Profit, and Passion. Rather than conceptualizing these domains in isolation, the ESRI frames them as interdependent value dimensions that may reinforce one another or generate tensions, depending on organizational priorities, stakeholder power, and contextual conditions.

In doing so, the ESRI enables a more integrated understanding of elite sport value creation and its associated tensions and trade-offs. It offers a way to examine how organizations and systems configure multiple value demands and how leadership and governance shape these configurations across contexts. Figure 1 presents the five dimensions as an interdependent configuration rather than a fixed hierarchy. The following sections elaborate each dimension in turn.

**Figure 1:**
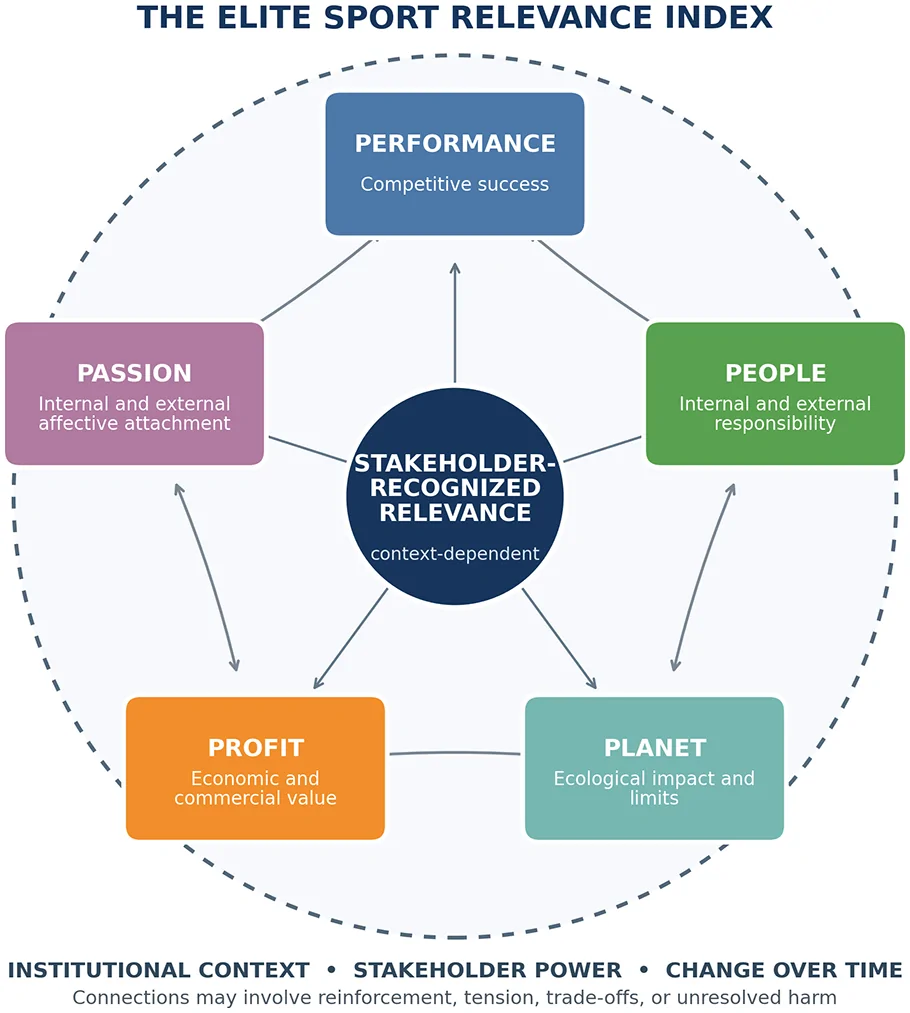
The Elite Sport Relevance Index (ESRI). The framework conceptualizes relevance as a stakeholder-recognized and context-dependent configuration of five interdependent value dimensions. Bidirectional connections indicate that relationships may involve reinforcement, tension, trade-offs, or unresolved harm. People comprises internal and external responsibility, while Passion comprises internal and external affective attachment. The surrounding layer indicates that configurations are shaped by institutional context, differences in stakeholder power, and change over time. Equal analytical inclusion does not imply equal empirical weight, ethical equivalence, compensability, or an expectation of balance. No empirical data are represented.

### 3.1 P1—performance: competitive success as organizational output

Performance constitutes a core and legitimate value in elite sport and functions as the primary organizational output around which elite sport systems and organizations are structured (32). Across national sport systems and elite sport organizations—such as clubs, federations, and leagues—performance is commonly operationalized through medals, rankings, championships, and qualification outcomes. These indicators provide clear, comparable, and communicable measures of achievement, making performance central to accountability, legitimacy, and strategic decision-making (1, 2).

At the organizational level, performance operates not merely as an outcome but as a dominant organizing logic. Elite sport organizations deliberately structure their strategies, governance arrangements, and professional practices around the production of competitive success. Investments in talent identification, athlete development pathways, coaching expertise, and performance support systems are oriented toward maximizing performance outputs (1, 2). In this sense, performance shapes organizational cultures and priorities, influencing how success is defined, how resources are allocated, and how organizational effectiveness is assessed. Performance thus functions as a structuring principle that permeates everyday practices within elite sport organizations.

At the level of national sport systems, performance is articulated through policy frameworks that emphasize results-based accountability and international comparison. Governments and sport agencies often justify public investment in elite sport by reference to measurable outcomes, particularly success at major international events. Although national ambitions vary—from medal dominance to representation at elite competitions—performance remains the primary metric through which systems communicate success internally and externally (3, 6).

Performance is also closely linked to broader processes of value creation in sport business and society. Sport activity provides the foundation for participation, engagement, and economic potential (15, 19). Elite performance is a highly visible and intensified form of that activity: it attracts attention, mobilizes audiences, and creates platforms through which economic, social, and symbolic value can be generated. Performance therefore underpins elite sport's capacity to function as a participation and engagement platform with potential physical, mental, social, and commercial outcomes.

At the same time, a performance-dominated environment creates risks. An excessive focus on results may encourage short-termism, ethical compromise, and the marginalization of human and societal considerations (5, 12). Performance pressure can shape expectations placed on athletes and staff and may undermine well-being, autonomy, and long-term development. When performance becomes the overriding criterion of success, other forms of value—such as good governance, social responsibility, and public legitimacy—risk being subordinated or instrumentalized.

### 3.2 P2—people: human-centered governance and social responsibility

The People dimension captures the human and social responsibilities of elite sport organizations. Within sport management scholarship, this dimension has traditionally been examined through the lens of corporate social responsibility (CSR), which addresses how sport organizations respond to societal expectations that extend beyond sporting and financial performance (33).

While CSR has provided an important foundation, more recent developments point toward broader frameworks of social accountability, such as Environmental, Social, and Governance (ESG) approaches, which emphasize the structural integration of social considerations into organizational strategy and governance (34). In elite sport contexts, this shift reflects increasing expectations that organizations not only contribute to society externally, but also take responsibility for the well-being, development, and fair treatment of their own athletes and staff.

#### 3.2.1 Internal responsibility: well-being, labor conditions, and organizational culture

A key distinction within the People dimension concerns external and internal social responsibility. Although much of the literature has focused on the contribution of elite sport organizations to community development, an equally critical aspect lies in their internal responsibility toward athletes and staff (33).

Internal responsibility encompasses the conditions under which individuals in elite sport organizations operate, including physical and mental well-being, labor conditions, organizational culture, and opportunities for personal and professional development. In high-performance environments, where performance pressures are structurally embedded, these conditions are a central governance concern. Elite sport organizations carry a duty of care to ensure that athletes and staff can perform in environments that are effective, safe, fair, and sustainable (33, 35).

Recent attention to athlete mental health and safeguarding has reinforced the urgency of embedding internal responsibility in governance structures. Performance cannot be detached from the human conditions under which it is produced (35). These concerns are ethical, but they are also linked to organizational effectiveness and legitimacy: organizations that fail to address internal responsibility risk undermining both their performance capacity and their societal standing.

Integrating people-related considerations into decision-making requires leadership that can govern tensions between performance imperatives and long-term human sustainability, often under conditions of uncertainty and competing pressures. From this perspective, value creation in elite sport is not only a matter of outputs, but also of how organizations structure and govern the processes through which those outputs are achieved.

#### 3.2.2 External responsibility: community engagement and social legitimacy

External responsibility within the People dimension is most visible in how elite sport organizations engage with surrounding communities. Sport management research has examined how clubs, leagues, and governing bodies use their visibility and cultural significance to contribute to health promotion, education, and social inclusion (14, 33). These activities reflect an expectation that elite sport organizations act as socially embedded institutions with responsibilities extending beyond competitive performance.

Such community-oriented practice describes structured efforts by sport organizations to address social issues through interventions and partnerships (14). These practices often involve collaboration with public and nonprofit actors and align with wider policy agendas related to public health, youth development, and social cohesion. External engagement is therefore both a societal commitment and a strategic activity through which organizations seek to strengthen legitimacy and stakeholder relationships.

At a conceptual level, external responsibility is a mechanism through which elite sport organizations generate and maintain social legitimacy. Their local roots, symbolic value, and visibility position them as potential vehicles for community development and social change (33). Through sustained engagement, sport organizations may contribute to social capital and collective identity, reinforcing their role as socially relevant actors.

However, the impact of community-oriented activity depends on the extent to which it is embedded in organizational strategy and governance rather than treated as a peripheral or symbolic initiative. When disconnected from core processes, such activity risks being perceived as instrumental or performative, undermining credibility and long-term value (14, 36).

From an ESRI perspective, external responsibility should therefore not be viewed in isolation, but in relation to internal responsibility. The credibility of external engagement is closely tied to the internal practices of sport organizations, particularly with regard to athlete and staff well-being, governance standards, and ethical conduct. Together, these dimensions shape how elite sport organizations create, communicate, and sustain social value across different contexts.

### 3.3 P3—planet: environmental sustainability and long-term legitimacy

The Planet dimension addresses the environmental responsibilities and ecological impacts of elite sport organizations and systems. In contrast to the People dimension, environmental sustainability has historically occupied a more marginal position in elite sport governance. However, recent literature indicates that Planet is becoming an increasingly salient value dimension, driven by climate change, sustainability transitions, and growing societal scrutiny of resource-intensive activities (13, 37).

At both organizational and system levels, elite sport contributes to negative environmental impact through travel-intensive competition calendars, energy consumption of venues, and the construction and maintenance of sport infrastructure. In other words, elite sport is not inherently climate-friendly, particularly in globalized competition formats that prioritize international mobility and scale (38–40). Yet, recent years have also seen positive contributions to the planet dimension: athletes have started speaking up about sustainability and decision-makers in sport are starting to indicate that climate change is an important issue to consider in elite sport programs (9). The literature increasingly emphasizes that environmental impact is shaped by how elite sport programs are structured and governed, rather than being an unavoidable by-product of sport (40).

The Planet dimension also introduces clear tensions with performance and commercial logics that prioritize expansion, visibility, and revenue growth. Elite sport organizations operate at the intersection of environmental and commercial demands, requiring explicit prioritization rather than assuming that these demands can be reconciled (40). Ignoring environmental considerations may weaken long-term legitimacy as stakeholders increasingly evaluate sport through an environmental lens (41), while prioritizing sustainability may challenge established performance and revenue models.

Illustrative examples include attempts to adjust international competition calendars, invest in lower-emission venues, and develop less resource-intensive event formats. Such interventions interact directly with commercial interests related to global reach, sponsorship value, and media exposure. They show that environmental sustainability is not only a constraint, but also a domain in which innovation and new forms of value creation may emerge.

From an ESRI perspective, the Planet dimension therefore functions both as a condition for long-term legitimacy and as a potential source of innovation. At the same time, these developments underline that environmental sustainability cannot be considered in isolation but must be understood in relation to the economic and commercial structures that underpin elite sport. This interdependence points directly to the role of Profit as a complementary and, at times, competing value dimension within elite sport systems.

### 3.4 P4—profit: economic value creation and the commercial turn

The Profit dimension captures the economic and commercial value created through elite sport. Within the ESRI framework, Profit refers to the financial resources, revenue models, and market-based mechanisms that enable elite sport organizations and systems to operate, professionalize, and sustain themselves over time. While elite sport has historically been embedded in public funding structures and voluntary traditions, sport management scholarship demonstrates a clear and sustained shift toward understanding sport as a commercial and managerial domain.

This shift can be traced back to the second half of the twentieth century, particularly in the United States, where sport became increasingly integrated into the structures of consumer capitalism and developed into a large-scale commercial enterprise (27). Over time, this model extended to European contexts and has since evolved into a global phenomenon, with emerging sport markets in regions such as the Middle East and Asia further accelerating the commercial transformation of elite sport (28).

At the organizational level, elite sport entities operate within complex commercial ecosystems, generating revenue through broadcasting rights, sponsorships, merchandising, ticketing, and digital platforms. Value is created through the interaction between sport activity, stakeholder engagement, and commercial opportunities (15).

Financial and sporting performance remain closely intertwined. Economic resources enable investment in talent development, infrastructure, and innovation, while sporting success enhances visibility, marketability, and revenue potential. Di Simone and Zanardi (42) show that financial and sporting performance can be mutually reinforcing rather than independent. This interdependence also introduces vulnerabilities: overreliance on specific revenue streams or results may expose organizations to financial and strategic instability. Diversification can therefore contribute to resilience and long-term sustainability (4, 29).

At the system level, Profit plays an equally central role. International federations, professional leagues, and event organizers rely heavily on commercial revenues to finance competitions, redistribute resources, and maintain organizational stability (43, 44). Mega-events and global competition formats further illustrate how elite sport systems are increasingly structured around commercial imperatives, with media exposure and market reach functioning as key drivers of value creation (45).

Within the ESRI framework, the Profit dimension thus reflects the structural embedding of elite sport within market-based and managerial logics. While economic value creation is essential for sustaining elite sport systems, its centrality also raises important questions about how financial objectives interact with other value dimensions. In particular, the commercialization of elite sport is closely linked to the generation of emotional engagement and fan identification, pointing to the role of Passion as a complementary and, at times, amplifying value dimension.

### 3.5 P5—passion: emotional and identity-based value

The fifth dimension of the ESRI framework is Passion. Sport is characterized by emotion, experience, and social attachment. Unlike many other industries, sport consumption is rarely driven by rational evaluation alone; it is intertwined with identity, belonging, and affect. These distinctive features make emotional and symbolic meaning central to how sport creates value (19). The distinction between People and Passion concerns the type of value being examined rather than the stakeholder group involved. People concerns the human conditions, rights, relationships, and social consequences through which elite sport is produced. Passion concerns the affective and symbolic attachments through which participation in elite sport becomes meaningful. The same person may therefore appear in both dimensions. Athlete well-being and labor conditions belong primarily to People, whereas an athlete's occupational identity, pride, or sense of calling belong to Passion. The dimensions can also conflict: strong occupational passion may enhance commitment and performance while simultaneously normalizing excessive workloads or precarious conditions under People.

#### 3.5.1 External passion: fan attachment, collective emotion, and cultural meaning

Within the sport management literature, Passion has been most extensively examined in relation to fans and spectators. Research on fan engagement demonstrates that emotional attachment to teams, athletes, or sports is a key driver of sustained involvement and behavioral engagement (46). Beyond identification with a team or club, this engagement often reflects a deeper sense of belonging and social connection, in which sport organizations function as sites of collective identity formation (47–49).

Wrexham AFC briefly illustrates how external Passion can interact with other dimensions. Through the documentary series *Welcome to Wrexham*, the club became a narrative-driven platform in which storytelling and identity formation extended local attachment to global audiences (50). This Passion may reinforce Profit through media, merchandise, and matchday demand. Yet global commercial expansion may also reshape the local attachments on which the narrative depends, showing that Passion can be both a source of value and an object of strategic appropriation.

#### 3.5.2 Internal passion: commitment, occupational identity, and calling

Passion is not confined to external stakeholders. Employees, coaches, and athletes also display strong affective commitment and identification with their organization and profession (51, 52). This motivation can be an organizational resource, contributing to commitment, creativity, and performance.

At the same time, passion is a double-edged dimension of value creation. Emotional engagement can strengthen commitment and performance, but it may also create vulnerability. Obsessive forms of passion and strong role identification can encourage overcommitment, blur boundaries between professional and personal life, and increase tolerance for demanding or precarious conditions (53). Passion may therefore enable value creation while obscuring structural challenges in elite sport systems.

From an ESRI perspective, Passion is therefore a foundational yet ambivalent dimension of elite sport value creation. It can amplify performance, commercial attractiveness, and social engagement, but it can also produce vulnerability, exploitation, or exclusion. Governance and leadership must recognize these effects and, where possible, mitigate them without presuming that every conflict can be resolved.

## 4 Discussion: governing interdependence and conflict across the five dimensions

Having introduced the five dimensions, the second part of this article shifts attention from individual value domains to their interaction. The value of the ESRI lies not in identifying five categories, but in making explicit the tensions, trade-offs, and choices between them. Elite sport organizations and systems continuously navigate multiple, interdependent dimensions that may reinforce one another or generate conflict over time. Understanding relevance therefore requires a dynamic view of how value is generated, how associated harms and costs are distributed, and how configurations are shaped by competing institutional logics (21–23).

The interaction between the five dimensions can take the form of reinforcement or tension. The ESRI does not presume that the five dimensions can or should reach equilibrium. Some complementarities can be fostered and some trade-offs can be mitigated, but other contradictions are persistent features of the institutional field. Responding to one institutional demand may require resisting another and may produce organizational paralysis or fragmentation rather than accommodation (54). Sustained sporting success may enhance fan engagement and emotional attachment, thereby increasing commercial value through sponsorship and media exposure. Socially responsible initiatives and community engagement may further strengthen organizational legitimacy, creating a reinforcing cycle across Performance, Passion, Profit, and People.

However, elite sport also provides numerous examples where value dimensions come into conflict. The organization of global sporting events, for instance, often illustrates tensions between commercial imperatives, environmental sustainability, and social responsibility. Efforts to maximize global reach, media exposure, and revenue generation may require extensive international travel, large-scale infrastructure development, and intensive resource use, raising concerns about environmental impact and long-term sustainability. Similarly, high-performance systems that prioritize medal success may place significant pressure on athletes, potentially conflicting with goals related to well-being and duty of care (9).

Because relevance is stakeholder-recognized, the analysis of a value configuration must identify whose evaluations count and why. Stakeholder judgments are neither equally weighted nor made in a neutral arena. Stakeholder salience is shaped by differences in power, legitimacy, and urgency, which influence which claims receive organizational attention (55). An ESRI analysis must therefore examine not only which stakeholders experience benefits, costs, or harm, but also which stakeholders control resources, possess decision rights, define performance criteria, and can hold the organization accountable. Relevance is therefore not the outcome of a neutral aggregation of stakeholder preferences. Actors controlling ownership, capital, media access, event rights, regulatory authority, or public funding can more readily translate their interests into formal organizational priorities. Athletes, workers, supporters, and local communities may possess moral legitimacy or urgent claims while lacking equivalent decision-making power. Capital concentration can consequently determine not only which dimension is prioritized, but also which benefits and harms become visible, measurable, or accepted (27, 55).

The extension of the Triple Bottom Line into the ESRI also changes the purpose and critical orientation of the original framework. Elkington (18) developed the Triple Bottom Line as a systemic challenge to conventional capitalism, rather than as a neutral instrument for balancing organizational objectives. He subsequently warned that the framework had been diluted through its use as an accounting and reporting device instead of a catalyst for systemic change (56). By transferring this architecture to elite sport governance and adding Performance and Passion, the ESRI becomes a more domain-specific, organizationally oriented framework. This increases its relevance to elite sport management, but it also attenuates part of the systemic critique embedded in Elkington's original formulation.

This shift has important consequences for how the ESRI should be interpreted. Giving Performance and Passion full analytical status creates a risk that sporting achievement or emotional resonance could be invoked to legitimate continued human, social, or environmental harm. Analytical inclusion does not, however, imply ethical equivalence or compensability. The ESRI is not an additive model in which exceptional performance, commercial revenue, or widespread emotional engagement can automatically offset serious harm under People or Planet. Its purpose is to expose such tensions and the priorities underlying them, not to demonstrate that they can invariably be balanced. Applying the ESRI therefore requires attention to who benefits, who bears the costs, which stakeholders possess decision-making power, and whether stated commitments are reflected in material practices such as budgets, incentives, competition calendars, and accountability arrangements.

### 4.1 Illustrative value configurations

The following case is not presented as an example of a dominant dimension or a harmonious configuration. It is used to examine how several dimensions become connected through organizational choices, how benefits and costs are distributed among stakeholders, and which tensions can be mitigated or remain unresolved. The analysis therefore considers the stakeholders involved and their relative power, relationships of reinforcement between dimensions, associated harms and costs, and the material practices through which particular priorities become dominant.

#### 4.1.1 The Dallas cowboys: commercial scale and its distributional and environmental trade-offs

The Dallas Cowboys illustrate how commercial scale can shape a contested elite sport value configuration. As one of the most valuable sport franchises globally, the organization's economic strength is not merely the result of market demand or brand equity; it is systematically produced through coordinated organizational structures, large-scale infrastructure, and integrated operational processes (57). Commercial value is thus a deliberately engineered outcome grounded in the club's capacity to deliver complex sport and entertainment experiences at scale.

The case demonstrates how commercial value is embedded in the operational architecture of elite sport. Matchday delivery at AT&T Stadium involves extensive logistical coordination across venue operations, fan services, infrastructure, and supporting systems, enabling the club to host audiences exceeding 90,000 spectators while generating substantial revenue streams from ticketing, hospitality, concessions, and ancillary services (57). The stadium itself functions as a multifunctional entertainment platform, extending beyond sport to host a wide range of events, thereby diversifying and stabilizing revenue streams. In this sense, Profit is not an isolated outcome but is structurally embedded in the design and execution of the sport product.

Commercial investment may reinforce other dimensions, but these effects are not automatic. Spending on infrastructure, service quality, and fan logistics can intensify external Passion through a highly curated matchday experience, while historical success connects commercial value to Performance even when on-field results fluctuate. Community-facing activities may contribute to People only when they produce demonstrable benefits and are aligned with credible internal labor and governance practices. Meanwhile, resource-intensive infrastructure and large-scale event operations distribute environmental and social costs among owners, workers, public authorities, and surrounding communities.

Viewed through the ESRI, the Dallas Cowboys represent a contested value configuration rather than a Profit-led success model. Commercial scale can support sporting investment and highly affective fan experiences, thereby connecting Profit, Performance, and Passion. At the same time, this model depends on resource-intensive infrastructure, continuous event consumption, and the distribution of operational costs among owners, workers, public authorities, and surrounding communities. The case therefore illustrates that reinforcing relationships between some dimensions may coexist with unresolved pressures in People and Planet. The relevant question is not whether the configuration is harmonious, but which benefits and harms it produces, who controls the configuration, and whose interests carry greatest weight.4.1.2 Olympic Mega-Events: Visibility, Public Investment, and Unequally Distributed CostsThe organization of global mega-events such as the Olympic Games provides a powerful illustration of how tensions between value dimensions are structurally embedded within elite sport systems. Although these events are frequently framed as catalysts for economic development, international prestige, and sporting excellence, a substantial body of research shows that their planning and delivery are characterized by recurring asymmetries and conflict. Müller (58) conceptualizes this as a “mega-event syndrome,” referring to interrelated dynamics that systematically produce adverse outcomes for host cities and societies.

From a Performance and Profit perspective, mega-events generate global visibility, commercial revenues, and opportunities for place branding. However, these anticipated benefits are often overstated, while costs are systematically underestimated, leading to significant budget overruns and inefficient allocation of resources. Empirical evidence shows that mega-events frequently involve large-scale public investments with limited long-term returns, raising fundamental questions about their economic rationale (58–60).

These dynamics directly impact the People and Planet dimensions. Mega-event planning tends to prioritize event-specific infrastructure over broader societal needs, often resulting in oversized or underutilized facilities, displacement of local communities, and increased socio-spatial inequality (61, 62). Environmental concerns are similarly pronounced, as large-scale construction, intensified mobility, and resource consumption contribute to significant ecological footprints. Rather than aligning with long-term sustainability goals, mega-events may therefore exacerbate environmental and social pressures.

A key mechanism underlying these tensions is what Müller (58) describes as “event takeover,” whereby the priorities of the event begin to dominate urban planning processes, effectively reversing the intended relationship between sport and development. In such cases, mega-events become drivers rather than instruments of policy, reshaping governance structures and reallocating public resources toward short-term visibility and delivery imperatives.

Viewed through the ESRI framework, mega-events exemplify how elite sport value creation can become skewed toward Performance and Profit, while generating significant trade-offs in the People and Planet dimensions. Importantly, these tensions are not incidental, but arise from the structural conditions under which mega-events are planned, governed, and delivered. As such, they highlight the inherent difficulty of aligning multiple value dimensions within large-scale, globally mediated sport systems. Mega-event configurations are produced through interactions among event-rights holders, host governments, organizing committees, sponsors, broadcasters, athletes, residents, workers, and civil-society organizations. These actors possess markedly unequal resources and decision rights. Event owners and host governments can institutionalize their priorities through contracts, budgets, and delivery deadlines, while local communities often have more limited influence despite bearing substantial social and environmental costs. The resulting configuration should therefore be understood as an outcome of stakeholder power and governance rather than neutral negotiation among equivalent interests.

### 4.2 Organizational logics, symbolic adoption, and governance

The preceding cases show that tensions and synergies between value dimensions are rooted in the coexistence of multiple institutional logics. Institutional logics are socially constructed systems of meaning that provide assumptions about appropriate behavior, legitimate action, and organizational success (63). In elite sport, they influence practices and organizational identity by making particular forms of conduct and value recognizable and legitimate. Yet, the five ESRI dimensions should not be treated as institutional logics in themselves. They are analytical domains of value that are interpreted and enacted through broader institutional logics, including competitive, market, state, professional, community, and environmental logics. More than one logic may shape a dimension, while the same logic may operate across several dimensions. Market logic, for example, influences Profit but may also commercialize Passion and reshape decisions concerning Performance and People.

Following Friedland and Alford (21), institutional logics consist of both symbolic constructions and material practices. Their coexistence does not imply that contradictions can be resolved through improved coordination. Conflicts between commercial expansion and ecological limits, or between intensive performance demands and durable athlete welfare, may be structural features of the interinstitutional field. The purpose of the ESRI is therefore to make such contradictions and their material consequences visible, including circumstances in which accommodation is partial, temporary, or impossible.

This identity dimension is important for understanding the five ESRI dimensions. Adopting Performance, People, Planet, Profit, and Passion as organizing dimensions implies more than expanding how an organization is assessed; it may require a shift in organizational self-understanding. An organization that takes People and Planet seriously cannot understand itself only as a producer of performance outcomes or a commercial platform. Likewise, integrating Profit and Passion into a public value proposition requires seeing the organization as part of an ecosystem of athletes, fans, communities, media, sponsors, governments, and markets. The five dimensions are thus both analytical categories and potential identity claims about what elite sport organizations are, what they stand for, and how they seek legitimacy.

Public and private value creation should therefore not be treated as separate domains, but as interdependent forms of value. Public–private partnerships can provide arrangements through which public institutions, commercial actors, sport organizations, media partners, and communities jointly configure value. Such partnerships do not automatically produce equitable or mutually compatible outcomes (25). As the mega-event case illustrates, hybrid arrangements may amplify asymmetries when commercial or political logics dominate social or environmental value. The key issue is how underlying logics are governed and translated into material commitments.

#### 4.2.1 Symbolic adoption and material practice

The ESRI is itself a symbolic construction and can be adopted ceremonially. An elite sport organization may publicly invoke Performance, People, Planet, Profit, and Passion while continuing to allocate budgets, authority, calendar space, and rewards almost exclusively to medals and commercial returns. In that situation, multidimensional language signals responsibility and legitimacy without changing the underlying hierarchy of priorities.

This possibility reflects institutional decoupling, whereby formal structures and public accounts can be separated from material organizational practice (64). Applying the ESRI therefore requires comparison between stated commitments and evidence in budgets, performance indicators, remuneration and incentives, procurement, competition calendars, athlete representation, environmental targets, and board accountability. Its diagnostic value lies partly in revealing where symbolic commitments remain weakly coupled to decisions and resource allocation.

#### 4.2.2 Leadership and governance

Leadership is crucial in this process. Selznick (65) conceptualizes institutional leadership as the work of value infusion: giving direction to the process through which an organization becomes meaningful and enduring. For elite sport leaders, the task is not only to optimize performance, revenue, or stakeholder satisfaction, but to decide which value commitments should become part of the organization's identity and how they are embedded in structures, routines, partnerships, narratives, and governance. This requires movement beyond individual leadership toward relational, collective, and system-level understandings of leadership in sport ecosystems (66–68).

This work requires more than generic management capability. Competency scholarship treats competencies as contextual, observable, and developable capabilities rather than static lists of personal attributes (69–72). Synthesizing this literature with the ESRI suggests four particularly relevant capacities: strategic sensemaking, adaptive judgment, relational governance, and self-regulation with integrity awareness. Together, these capacities help leaders interpret changing expectations, make choices when values conflict, build stakeholder alignment, and protect integrity under performance, financial, or political pressure.

The leadership challenge is therefore not only to recognize the five value dimensions, but to govern their interaction. Many ESRI tensions are not technical problems, but judgment problems. Choices between medals and well-being, growth and sustainability, autonomy and system coordination, or short-term performance and long-term legitimacy cannot be solved by metrics alone. They require leaders who can make trade-offs explicit, explain why certain choices are made, and embed these choices in strategy, resource allocation, governance routines, partnerships, and accountability structures. Leadership can alter organizational practices, redistribute voice, and strengthen accountability, but it cannot eliminate ecological limits, unequal power, or every structural conflict between the five dimensions.

This implies a shift from performance leadership to value-configuring leadership. Elite sport leaders must ask not only whether the organization is successful, but what kind of success is being produced, at what cost, for whom, and over what time horizon. They need to identify when one value logic is becoming too dominant, when important forms of value are being crowded out, and when the organization's legitimacy depends on revising its priorities. Recent sport leadership development research suggests that such capacities develop over time through experience, relationships, reflection, and situated practice rather than through individual training alone (73).

#### 4.2.3 Applying the ESRI in practice

Applied as a diagnostic and deliberative framework, the ESRI involves six connected tasks: defining the organizational or system boundary; identifying stakeholders, claims, and differences in power; examining value creation, harms, and costs under each dimension; identifying reinforcement, conflict, and accepted or unavoidable sacrifices; comparing stated commitments with budgets, indicators, incentives, decision rights, competition calendars, and accountability arrangements; and revisiting the configuration over time. These tasks do not yield a single optimal score. They structure evidence-based deliberation and make priorities, exclusions, and responsibilities explicit for boards, directors, federations, funders, athletes, commercial partners, and performance teams.

## 5 Conclusion and future research agenda

The ESRI reframes elite sport relevance as an ongoing governance challenge rather than a fixed outcome. Performance remains indispensable, but it becomes socially durable only when considered alongside the human, environmental, economic, and emotional values captured by People, Planet, Profit, and Passion. The framework's main contribution is therefore not the addition of four objectives to performance, but a vocabulary for making visible the complementarities, tensions, and trade-offs among value dimensions shaped by competing institutional logics. Leadership and governance mediate how those tensions are interpreted and embedded in strategy, resource allocation, partnerships, and accountability, but they cannot dissolve every structural conflict.

### 5.1 Limitations and future research

A limitation is that the ESRI is itself a symbolic construction. Its terminology may be used to signal multidimensional responsibility while the underlying distribution of resources, authority, and rewards remains largely unchanged. The framework cannot prevent such ceremonial adoption. Empirical applications must therefore examine the degree of coupling between stated commitments and material practices and identify which dimensions continue to dominate organizational decision-making. Future research should study when ESRI-related language changes practices, when it remains symbolic, and which governance arrangements strengthen substantive accountability.

Another limitation lies in the illustrative cases, which are drawn predominantly from mature, highly visible, and commercialized sport settings. They cannot establish that the framework operates identically in less-commercialized sports, resource-constrained federations, or different political and cultural contexts. In such settings, Profit may primarily concern financial viability rather than commercial growth, while Passion may be expressed through local rather than global attachment. Comparative research should therefore examine whether all five dimensions are recognizable across settings, how their meanings and salience vary, and whether additional or alternative dimensions are required.

The immediate research agenda is to move from conceptual specification to empirical testing. Priorities are to operationalize each dimension; examine whether stakeholder groups interpret and weight the dimensions differently; compare configurations across sports, countries, and organizational levels; and study how leadership and governance practices alter those configurations over time. Longitudinal research should examine which configurations emerge, whose priorities shape them, who benefits, which harms remain, and when multidimensional commitments become coupled to material organizational practice.

For practice, the ESRI offers boards, federations, funders, athletes, and commercial partners a structured basis for asking what kind of success they are producing, for whom, at what cost, and over what time horizon. Elite sport's future relevance will depend not only on its capacity to win, but on its ability to create value that stakeholders continue to regard as meaningful, legitimate, and sustainable.
